# Risk based culling for highly infectious diseases of livestock

**DOI:** 10.1186/1297-9716-42-81

**Published:** 2011-06-29

**Authors:** Dennis E te Beest, Thomas J Hagenaars, J Arjan Stegeman, Marion PG Koopmans, Michiel van Boven

**Affiliations:** 1 Department of Farm Animal Health, Faculty of Veterinary Medicine, Utrecht University, Yalelaan 7, 3584 CL, Utrecht, The Netherlands; 2 National Institute for Public Health and the Environment (RIVM), Antonie van Leeuwenhoeklaan 9, 3721 MA, Bilthoven, The Netherlands; 3 Quantitative Veterinary Epidemiology, Animal Science Group, Wageningen UR, P.O. Box 65, 8200 AB, Lelystad, The Netherlands

## Abstract

The control of highly infectious diseases of livestock such as classical swine fever, foot-and-mouth disease, and avian influenza is fraught with ethical, economic, and public health dilemmas. Attempts to control outbreaks of these pathogens rely on massive culling of infected farms, and farms deemed to be at risk of infection. Conventional approaches usually involve the preventive culling of all farms within a certain radius of an infected farm. Here we propose a novel culling strategy that is based on the idea that farms that have the highest expected number of secondary infections should be culled first. We show that, in comparison with conventional approaches (ring culling), our new method of risk based culling can reduce the total number of farms that need to be culled, the number of culled infected farms (and thus the expected number of human infections in case of a zoonosis), and the duration of the epidemic. Our novel risk based culling strategy requires three pieces of information, *viz*. the location of all farms in the area at risk, the moments when infected farms are detected, and an estimate of the distance-dependent probability of transmission.

## Introduction

Epidemics of infectious diseases such as classical swine fever, food-and-mouth, and avian influenza continue to wreak havoc in commercial livestock [1-6]. Efforts to control such outbreaks rely heavily on culling of infected farms, and farms in the vicinity of infected farms. This approach induces massive economic costs and leads to great animal suffering. It is therefore desirable to make as efficient as possible use of the available resources, and to spare as many animals as possible. Furthermore, in case of diseases that have zoonotic potential, such as highly pathogenic avian influenza A viruses of the H5 and H7 subtypes, it is also important to minimize the risk of human exposure [7-11].

The aim of preventive culling in outbreaks of commercial livestock is to contain the epidemic by removing susceptible flocks in the vicinity of infected farms; a typical strategy used for this is ring culling. In this strategy all farms within a certain radius of an infected farm are culled, typically starting close to the infected farm(s). The distance to an infected farm is related to the probability of a farm becoming infected. Ring culling therefore essentially involves culling the farms with the highest probability of becoming infected. We argue that, in addition to the distance to infected farms, another factor that is important is the local density of neighbouring farms. The local density determines how an epidemic is likely to develop, e.g.: a farm in an area with high density will likely cause more new infections than a farm in area with a low density; this implicitly follows from the relationship between distance and risk. In previous work the number of new infections that each infected farm is expected to cause was quantified by a farm reproduction number (R) and they were used to create risk maps that indicated areas with potential for high epidemic spread [12].

In this paper a novel culling strategy is introduced that takes into account not only the distance of susceptible farms to the infected farms, but also the number of secondary infections that a susceptible farm is expected to produce should it become infected. Specifically, we calculated for each farm not yet (known to be) infected a so-called risk value, which represents the number of infections the farm is expected to produce given current information on the unfolding of the epidemic. We argue that farms which rank highest in the risk based ordering should be culled first, thereby achieving an efficient allocation of resources (i.e. time, money, equipment). In practice, the risk value of each susceptible farm is given by the probability that a farm will become infected in a certain time span multiplied by the reproduction number of the farm once it is infected. Similar ideas that incorporate the connectivity of farms or individuals in applying an intervention measure have been suggested before in (non-spatial) network models of infectious disease spread [13]. For example by vaccinating friends' of friends which proved to be more effective than vaccinating people at random [14]. Similar is also the strategy to vaccinate children with the aim to reduce disease transmission [15] or to preferentially vaccinate large urban centres to reduce their role as disease reservoirs [16].

Our risk based culling scheme works with three pieces of information. First the locations of all farms in the area at risk need to be known. Second, an assessment of the current state of the epidemic should be available, in particular which farms are infected, and which farms are still susceptible. Third, an estimate of how the transmission probability depends on the distance between infected and susceptible farms should be at hand. The first two pieces are usually readily available during an epidemic. For the third piece of information estimates from past epidemics can usually be used [1,4,12,17].

We evaluate the performance of the risk based culling strategy in a simulation study that is loosely based on a large outbreak of highly pathogenic H7N7 avian influenza in the Netherlands. Parameter values and the transmission hazard are based on experience with this outbreak [6,12]. Throughout we systematically compare the effectiveness of our risk based culling strategy with the traditional approach that relies on the culling of farms in a ring (1-3 km) around infected premises. The comparisons are based on (1) the number of infected farms culled, which is related to the expected number of human infections, (2) the duration of the epidemic, and (3), the total number of farms culled.

## Materials and methods

### Modelling

The spread between farms was modelled with a stochastic SEIR model (Susceptible-Exposed-Infected-Removed) that operates with fixed time steps of 1 day. The probability *q_i _*that a susceptible farm *i *is infected on a particular day *t *is given by:(1)

where *λ_i_(t)*, the force of infection on farm *i *at day *t*, is calculated according to:(2)

**Figure 1:**
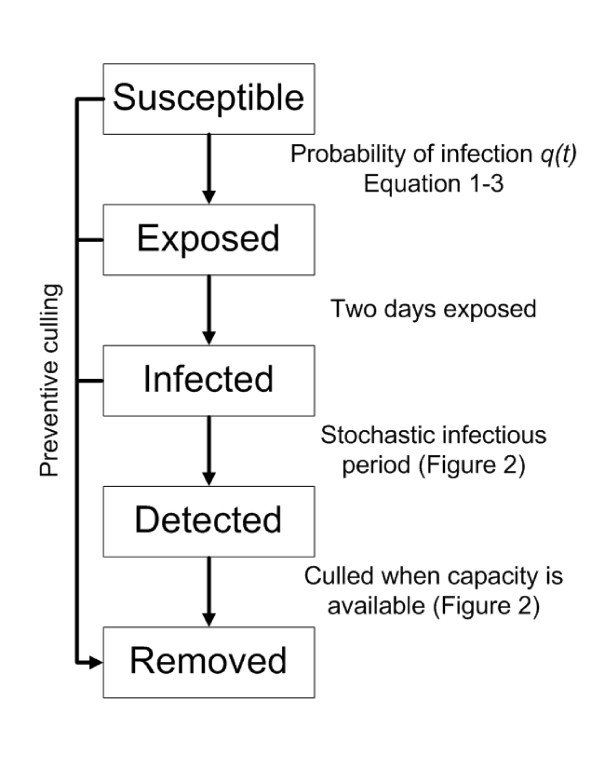
**Classification of the farms in the model**. Farms are either susceptible to infection, infected but not yet infectious (exposed), infected and infectious, detected and not infectious anymore, or removed from the system by culling.

The function *h(r_ij_) *is called the transmission kernel. It is defined as the infection hazard posed by farm *j *to farm *i *as a function of the inter-farm distance *r_ij _*[12], 1 is an indicator function that is 1 if j is infected (Figure 1) and 0 otherwise. In equation 2 it is assumed that transmission between farms is distance dependent, which for many outbreaks provided a satisfactory description of the data [1,4,12,17]. A number of mechanisms may be able to cause spread from farm to farm. Virus may for example be carried over by people, vehicles, or wind. In our model we adopt a phenomenological approach, and do not explicitly model different transmission mechanisms.

Although it is our goal to investigate the efficiency of risk based culling strategies in general, the parameters are specifically tailored to mimic the spatial spread of highly pathogenic avian influenza viruses in densely populated poultry areas. The shape of the hazard and estimates of the parameters to scale the hazard were estimated from the outbreak of highly pathogenic H7N7 avian influenza in the Netherlands [12]. The shape that fitted best was of the form:(3)

where *h_0_, r_0_*, and *α *are parameters of the hazard function (Table 1), and *r *is the distance between farms.

**Table 1:** Settings of the hazard kernel (equation 3) as used in the base scenario and the scenarios of the sensitivity analyses

Base hazard kernel	h_0_	0.0016
	r_0_	1.9
	α	2.1
Increased R0	h_0_	0.0020

Decreased R0	h_0_	0.0012

Increased tail	h_0_	0.0009
	r_0_	1.9
	α	1.4

Decreased tail	h_0_	0.0023
	r_0_	1.9
	A	2.8

Increased clustering	h_0_	0.0012

Decreased clustering	h_0_	0.0020

Misspecified kernel		0.00028

Only the modifications of the base scenario are shown. For the scenario with the misspecified kernel, the hazard is constant.

**Figure 2:**
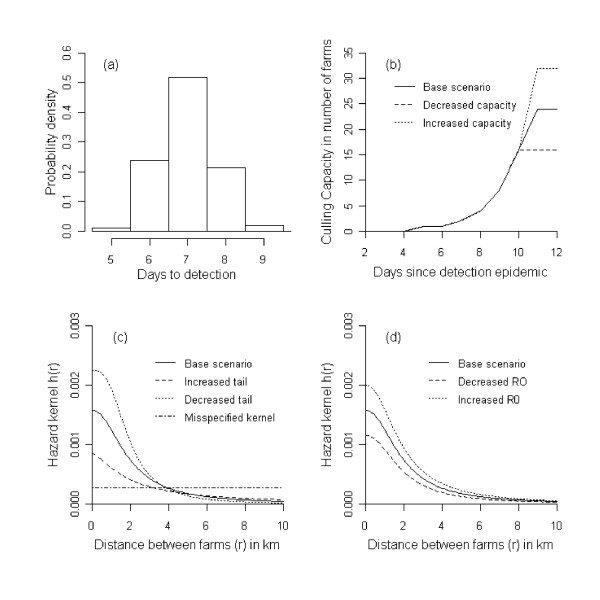
**Overview of model parameters**. (a) Distribution of the days to detection of an infected farm, (b) Culling capacity as a function of the time since detection of the outbreak, (c) Hazard kernels with an increased and decreased tail, and the misspecified kernel, (d) Hazard kernels with increased and decreased capacity.

We assume that upon infection each farm first becomes exposed (i.e. infected but not yet infectious) for a period of two days (Figure 1) [12,18]. After the exposed period has elapsed the farm is assumed to be infectious until it is detected. Here we assume for simplicity that upon detection the farm immediately ceases to be infectious to other farms, e.g., due to appropriate biosafety measures. In our simulation the time between becoming infectious to becoming detected is drawn from a gamma distribution with a mean (*T*) of 7 days and a shape parameter (*c*) of 100 (Figures 1 and 2a). Culling in the model is done (as in reality) with limited daily capacity. Farms that are culled are removed from the system, and cease to play a role in the infection dynamics. The culling capacity is assumed to be low at start of the epidemic and then increases quickly reaching a maximum after 11 days (Figure 2b) and is based on the situation of 2003 [6]. We assume that culling of detected infected farms is given priority above preventive culling. Each time step all detected infected farms are therefore culled first providing capacity is available (Figure 1 and Figure 2b). If the number of infected farms is larger than the culling capacity on a particular day then the infected farms detected last are culled the next day(s). If the culling capacity is greater than the number of detected infected farms then the remaining culling capacity is used for preventive culling. Preventively culled farms, whether they were susceptible, exposed or infected, become removed (Figure 1).

### Culling strategies

We now define in more detail the two culling strategies considered in this paper: ring culling and risk based culling. With ring culling all farms within a certain radius of any of the farms where an infection was detected are preventively culled. Culling is then continued until there are no more farms present in any of the (possibly overlapping) rings around infected farms. Ring culling is typically carried out inside-out, i.e. starting near the infected farm on the inside of the ring. In our model this is mimicked by consistently selecting the farm that is closest to a farm where an infection has been detected. An alternative ring culling strategy works from the outside of the ring to the inside. The rationale for this strategy is that it may help contain the infection within the culling ring. In this strategy the farm that is furthest away (but within the ring) of any farm where an infection was detected is culled first. In The Netherlands in 2003 culling was started on the inside of the ring close to the infected farms. At the start of the epidemic a 1 km ring was used, and later in the epidemic 3 km rings were used. In our analysis we consider both these radii. In our calculations for outside-in ring culling we considered a scenario with a ring radius of 3 km.

With risk based culling the estimated number of infections each farm is expected to create is used as culling criterion. The candidate farm with the highest expected number of new infections is then preventively culled first. The expected number of infections *E_i _*for farm *i *is given by the product of the probability that farm *i *is infected (*q_i_*, equation 1) and its reproduction number (*R_i_*, equation 7):(6)

Assuming a gamma distributed infectious period with mean *T *and a shape parameter (*c*) of 100, the reproduction value (*R_i_*) is calculated for each remaining farm (not detected, not culled), only taking into account all other remaining farms, as follows [12]:(7)

where *i *and *j *run through all farms that are not detected and that not have been culled.

**Figure 3:**
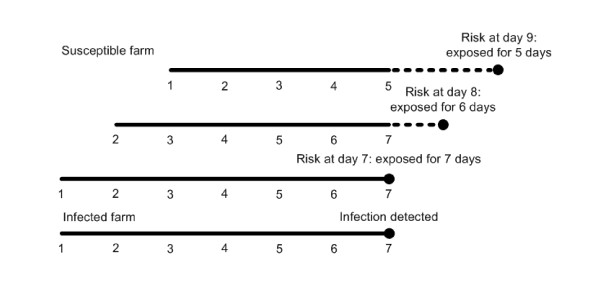
**Overview of how to calculate the approximate risk that farms were infected in the past based on knowledge of the detected infections (equation 8)**. In the example below, at day 7 an infected farm is identified as being infected. On day 7 a neighboring farm has on average been exposed to the infected farm for the last 7 days. If the susceptible farm was not detected as being infected then on day 8, it has been on average exposed for 6 days. And so on, until day 14 when on average there is no further exposure. In equation 5 at, for example, day 9, t = 9, t_jd _= 7, and T = 7 which results in 5 days exposure.

The exact probability for a farm to become infected (*q_i_*, equation 1) can in practise not be calculated during the epidemic as this would require complete knowledge on which farms are currently infected (also the ones that are yet undetected). However, it is possible to approximately calculate the risk that farms were infected in the past based on knowledge of the detected infections. In this calculation, we first assume that all detected farms have been infectious for exactly *T *days (mean infectious period, seven days). We note that this assumption could be dropped if detailed knowledge on the infectious period of the source were available during the epidemic. Then we approximate how much exposure each farm has had in the past *T *days. Suppose one particular farm *j *is detected as infected at time *t_jd_*. This means that all neighbouring farms have been exposed to this infected farm for the previous *T *days. If on one particular neighbouring farm no infection is detected up until *x *days after the detection of its infected neighbour (with *x *smaller than or equal to *T*) it should have escaped infection by that infected neighbour for at least *T-x *days (Figure 3). In agreement with this we estimate an approximate cumulative hazard λ_i_* of infection for each farm *i *where no infection was detected up until time *t *according to:(8)

The hazard is approximate (as indicated with the *) because farms that are infected but have not been yet been detected are not taken into account because these are unknown at that point in time. The approximate probability (q_i_*) that farm *i *was infected in the past based can be calculated according to(9)

This probability is used to calculate the approximate expected number of infections caused by farm *i*:(10)

 is used to rank the farms according to risk in order to determine the order for risk based preventive culling. In our model we used a risk threshold ("thr") below which farms are not culled anymore. In our model calculations we analyze the sensitivity of our results to changes in the threshold value.

### Simulation details

The efficiency of various conventional ring culling strategies and the novel risk based culling strategy were assessed using simulations of outbreaks on maps with randomly generated farm locations (see Additional file 1, Supplementary text and Additional file 2, Figure S1). Each map consisted of a circular inner area of 1000 farms with a density of 3.8 farms per m2 and an outer (ring-shaped) area of 1000 farms with a density of 0.5 farms per m2. The density assumed in the inner area is equal to the density in the largest poultry area of the Netherlands which is where most of the 2003 epidemic of avian influenza occurred. The density of the outer area is the density observed in the remainder of The Netherlands.

**Figure 4:**
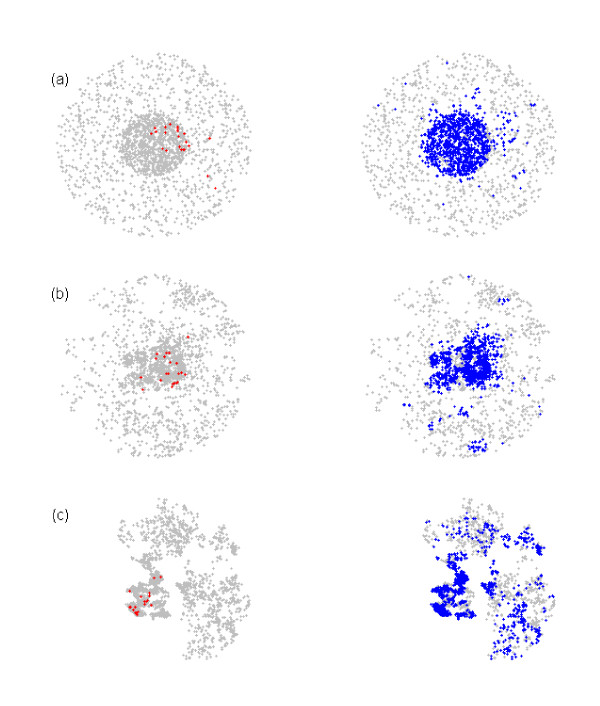
**Examples of outbreaks (a) homogeneous Poisson clustered, (b) moderately clustered (base scenario), and (c) clustered map**. Left graph shows a starting position with infected farms in red, the right graph shows an end situation with culled farms in blue.

To prevent early stochastic fade-out, and to condition on a large epidemic, all simulations were seeded with 10 infectious and 10 exposed farms (Figure 4). The starting configuration of the 10 infectious and 10 exposed farms was itself created with the transmission model (equations 1-3 and Figure 1). The first infected farm was randomly selected. The start-up simulation was run until 10 farms were infectious and 10 farms were exposed. In this manner we generated 50 different random starting configurations. For each culling scenario considered we did 400 simulations in which we used each map 8 times. For base-line values for the kernel parameters (Table 1) we carried out 2000 additional simulations (40 per map) for three of the culling strategies to evaluate the performance of these culling strategies per map.

In a future epidemic the exact shape of the hazard kernel of the disease spread is likely to be unknown. We therefore tested the sensitivity of the results to changes in the hazard kernel (Table 1), while the kernel to calculate the risk as a basis for preventive culling was left unchanged. Specifically, we considered two additional scenarios, one with a heavy-tailed kernel, and one in which the tail of the kernel falls of more quickly than in the default scenario (Figure 2d). We also investigated a situation with a more extensive difference between the hazard kernel used to calculate the disease risk and the actual hazard kernel of the disease spread (i.e. a misspecified kernel). For this we used a kernel that has equal disease spread disease up to 10 km (Figure 2c). The hazard kernels were adjusted so that the average reproduction number across all maps was the same for all three scenarios (i.e. *R *= 1.65) (Table 1). We furthermore considered scenarios with a high (*R *= 2.1) and low (*R *= 1.2) reproduction number (Table 1 and Figure 2d). Finally, in the base scenario the maximum culling capacity was fixed at 24 farms per day. To investigate the robustness of our results to the exact culling capacity we investigated scenarios with an increased (32 farms per day) or decreased (16 farms per day) culling capacity.

The relevant outputs of the model are the total number of farms culled, the number of infectious farms culled, and the duration of the epidemic (defined as the time from the first detection until the last culling of an infected farm). The total number of farms that are culled and the duration of the epidemic are both measures with economic relevance, not only because of the direct costs of culling, but also because under EU regulations borders will be closed for export during (an for some period after) an epidemic of avian influenza. Furthermore, minimizing animal suffering in itself is a worthy goal. The number of farms that are culled while being infectious is relevant because it determines the level of human exposure to an agent with zoonotic potential [7-11].

The simulation results obtained contain three sources of variation, i.e. (1) the culling strategy, (2) the random maps, and (3) the stochastic epidemic process. To separate these variances, and to single out the effect of the culling strategy, we analyzed the simulation results with a linear mixed model that used the maps as a random effect and the culling strategy as a fixed effect. The mixed model was used to estimate confidence bounds for the effect of the various culling strategies.

## Results

Risk based culling reduced the number of infected farms culled compared to both 1 and 3 km ring culling strategies, and thereby should be able to reduce the number of human infections (Table 2). Of three criteria by which we tested the control strategies, (1) number of infected farms culled, (2) total number of farms culled, and (3) duration of the epidemic, risk based culling outperformed ring culling always on two out of three criteria. On the third criterion it typically performs either equally well or slightly better (Tables 2 and 3). Risk based culling has a much lower number of infected and total number of farms culled compared to 3 km ring culling, and usually shortens the length of the epidemic (depending on the risk threshold). Compared to 1 km ring culling, risk based culling has a lower number of infected farms culled, and the duration of the epidemic is shorter. The total number of farms culled is either equal or lower (depending on the risk threshold).

**Table 2:** Simulation results for the various risk based and ring culling strategies in the base scenario

Culling strategy	Culled infected		Total farms culled		Epidemic in days	
Risk based, thresh = 0.001	216	(194;239)	958	(920;971)	57	(58;59)
Risk based, thresh = 0.0005	-*3*	(-*11;5)*	*+114*	*(100;128)*	-*3*	(-*4;*-*2)*
Risk based, thresh = 0.005	*+3*	(-*5;11)*	-*72*	(-*86;*-*58)*	*+3*	*(2;4)*
Risk based, thresh = 0.001/3 km ring^1)^	*+5*	(-*3;13)*	*+82*	*(68;96)*	-*2*	(-*3;*-*1)*
Ring 1 km, In - > Out ^2)^	*+32*	*(24;40)*	*+125*	*(111;139)*	*+4*	*(3;5)*
Ring 3 km, In - > Out ^3)^	*+27*	*(19;35)*	*+597*	*(583;611)*	*+0*	(-*1;1)*
Ring 3 km, Out - > In	*+111*	*(103;119)*	*+674*	*(660;688)*	*+7*	*(6;8)*

Brackets indicate 95% confidence interval.

The results of "Risk based, thresh = 0.001" with confidence bounds were estimated as the intercept of a mixed model that incorporated maps as a random effect and culling strategy as a fixed effect. The "+" or "-" for the alternative strategies indicate the difference compared to "Risk based, thresh = 0.001". Confidence bounds for the alternative strategies are also around the difference.

^1) ^Risk based culling in a 3 km ring.

^2) ^In - > Out: culling starts with farms that are closest to the infected farm.

^3) ^Out - > In: culling starts with farms within the ring that are farthest from the infected farm.

**Table 3:** Overview of results obtained with various scenarios used in the sensitivity analysis

Scenario	Culling strategy	Culled infected		Total farms culled		Epidemic in days	
Decreased capacity	Risk, thresh = 0.001	374	(329;420)	996	(964;1027)	72	(71;74)
	Ring 1 km, In - > Out ^1)^	*+46*	*(32;60)*	*+223*	*(209;237)*	*+4*	*(3;5)*
	Ring 3 km, In - > Out ^2)^	*+57*	*(43;71)*	*+704*	*(690;718)*	*+4*	*(3;5)*

Increased capacity	Risk, thresh = 0.001	161	(145;176)	1064	(1038;1090)	47	(46;47)
	Ring 1 km, In - > Out	*+20*	*(15;25)*	*-70*	*(-83;-56)*	*+8*	*(7;9)*
	Ring 3 km, In - > Out	*+16*	*(11;21)*	*+410*	*(396;423)*	*+1*	*(0;2)*

Increased R0	Risk, thresh = 0.001	460	(412;507)	1250	(1224;1276)	65	(64;66)
	Ring 1 km, In - > Out	*+64*	*(50;77)*	*+108*	*(98;119)*	*+5*	*(4;6)*
	Ring 3 km, In - > Out	*+61*	*(47;74)*	*+543*	*(532;553)*	*+2*	*(1;3)*

Decreased R0	Risk, thresh = 0.001	100	(91;110)	798	(771;825)	44	(43;45)
	Ring 1 km, In - > Out	*+13*	*(9;16)*	*+4*	*(-12;20)*	*+7*	*(6;9)*
	Ring 3 km, In - > Out	*+14*	*(10;17)*	*+509*	*(493;525)*	*+3*	*(1;4)*

Increased tail	Risk, thresh = 0.001	183	(169;197)	1076	(1050;1103)	59	(58;60)
	Ring 1 km, In - > Out	*+23*	*(16;30)*	*+40*	*(25;55)*	*+5*	*(4;6)*
	Ring 3 km, In - > Out	*+18*	*(11;25)*	*+632*	*(617;647)*	*+2*	*(1;3)*

Decreased tail	Risk, thresh = 0.001	220	193;247	958	(986;929)	50	(49;51)
	Ring 1 km, In - > Out	*+35*	*(27;42)*	*+34*	*(19;48)*	*+7*	*(6;8)*
	Ring 3 km, In - > Out	*+29*	*(21;36)*	*+396*	*(381;411)*	*+3*	*(2;4)*

Less clustering	Risk, thresh = 0.001	191	(175;206)	1003	(983;1023)	55	(54;56)
	Ring 1 km, In - > Out	*+33*	*(23;44)*	*-14*	*(-32;4)*	*+8*	*(7;10)*
	Ring 3 km, In - > Out	*+31*	*(21;42)*	*+443*	*(426;461)*	*+3*	*(1;4)*
More clustering	Risk, thresh = 0.001	277	(222;332)	1127	(1079;1176)	57	(55;59)
	Ring 1 km, In - > Out	*+43*	*(31;55)*	*+186*	*(163;210)*	*+6*	*(4;7)*
	Ring 3 km, In - > Out	*+38*	*(26;50)*	*+624*	*(601;648)*	*+4*	*(3;5)*

Misspecified kernel	Risk, thresh = 0.001	255	*(231;278)*	1095	*(1069;1121)*	59	*(58;60)*
	Ring 1 km, In - > Out	+24	*(15;32)*	+19	*(6;32)*	+4	*(3;5)*
	Ring 3 km, In - > Out	+25	*(16;33)*	+397	*(384;410)*	+1	*(0;2)*

Brackets indicate 95% confidence interval.

The results of "Risk based, thresh = 0.001" with confidence bounds were estimated as the intercept of a mixed model that incorporated maps as a random effect and culling strategy as a fixed effect. The "+" or "-" for 1 km and 3 km ring culling indicate the difference compared to "Risk based, thresh = 0.001". Confidence bounds for 1 km and 3 km ring are also around the difference.

^1) ^In - > Out: culling starts with farms that are closest to the infected farm.

^2) ^Out - > In: culling starts with farms within the ring that are farthest from the infected farm.

There was substantial variation between the maps. Per map the relative improvement of risk based culling over ring culling remained approximately the same. The mean number of infected farms per map culled (across the three main strategies) ranged from 82 to 474, the total number of farms culled ranged from 956 to 1423, and the duration of the epidemic ranged from 53 to 64. The performance of each strategy per map was studied by carrying out an additional 2000 simulations per strategy, i.e. 40 for each map. On 6 out of 50 maps 1 km ring culling had a lower total number of farms culled than risk based culling, and 3 km ring culling gave a shorter epidemic on 5 maps. With the exception of these, risk based culling consistently outperformed 1 km and 3 km ring culling.

In the sensitivity analyses we investigated the effect of changes in culling capacity, reproduction number, tail of the hazard kernel, the level of clustering of farms, and a misspecified hazard kernel (Table 3). Qualitatively, the results remained the same in all sensitivity analyses. In all scenarios risk based culling outperformed ring culling on two or three criteria. Quantitatively, the total or infected number of farms culled, and the length of the epidemic varied greatly between the scenarios. The relative improvement of risk based culling over ring culling, however, remained approximately the same.

## Discussion

Preventive culling of farms is an important control measure to halt epidemics of highly infectious diseases of livestock such as classical swine fever, foot-and-mouth disease, and avian influenza. This paper introduces a novel prioritization scheme for culling of farms that is based on the idea that farms with the highest expected number of secondary infections should be culled first. Our simulations show that risk based culling outperforms ring culling in terms of the number of infected farms culled, the total number of farms culled, and the duration of the epidemic. As risk based culling reduced the number of infected farms that are culled it is therefore also expected to reduce the number of human infections. We find substantial variation in the outcome between different maps but for a given map risk based culling consistently outperformed ring culling. This indicates that the spatial structure has a large influence on the outcome of an epidemic, which is supported by previous research [4,17,19-21].

Although the model presented here is parameterised for the avian influenza epidemic that occurred in The Netherlands in 2003, the methodology of risk based culling is more generally applicable to other infectious diseases controlled by culling. The only information needed are the locations of the farms, the moments at which infected farms were culled (both essential for any control and usually available from surveillance), and an estimate of the distance-dependent probability of transmission. An extension of our method that could potentially further improve risk based culling would be to not only focus on the expected number of infections within one infection generation, but try to estimate the expected number of infections in second and perhaps even third infection generations in the future.

We assumed that all farms are equally infectious which is reasonable for avian influenza [12] but for other diseases this may be different. Variability in susceptibility and infectivity can also be taken into account in risk based culling, proving estimates are available. One example where such variability existed is foot-and-mouth outbreak in the UK. For this epidemic a model was derived that is similar to the model used in this paper [3,4,17] and it can be used to calculate the probability of infection and reproduction number per farm as needed for risk based culling.

It is possible with an extensive misspecification in, for example, the infectivity of farms that risk based culling would be less effective. Note though that alternative strategies (such as ring culling) may suffer similarly. The challenge here is to have a good epidemiological understanding of how a disease spreads and incorporate this knowledge into the calculations. We believe that if misspecifications are minor, the reproductive number still identifies patches of farms that are close together weighted by their distance to infected farms. Quantitatively the outcomes may differ to some extent but qualitatively (risk based culling is about prioritizing) they may still be accurate. The effectiveness of risk based culling also depends on the culling capacity relative to the spread of disease. If the culling capacity is too low any control is impossible. If the culling capacity is very high then the order of culling becomes irrelevant. In between these extremes, culling resources need to be used efficiently and risk based culling can aid in this.

One advantage of risk based culling is that it does not require a certain arbitrary ring to be set. It can be argued however that the threshold needed in risk based culling to set the minimum risk level for culling is also arbitrary, and there is indeed not one clear risk based threshold (*thr*) that achieves the best results across all three criteria. If the threshold is decreased, the number of farms culled is increased and the pool of susceptible farms depletes quicker, which means an epidemic is stopped earlier. An epidemic that stops more rapidly is likely going to have less infected farms culled, and thus less human infections. Vice versa, if the threshold is increased, the total number of farms culled decreases but the length of the epidemic and the number of infected farms culled increase. Which strategy is best thus depends what goals decision makers want to achieve. Economically the cost of the total number of farms culled and the cost of a longer epidemic can be weighted. The impact on public health (human casualties) is however difficult to weigh and are dependent on the disease. With a disease like avian influenza which has a clear zoonotic potential reducing the public health impact is arguably the most important.

For policy makers our risk based culling policy may be more difficult to justify to stake holders and the public than the simple traditional ring culling strategy. In addition, to be acceptable any culling strategy would have to satisfy the requirements of regulatory bodies. An intuitively appealing strategy may be to apply a risk based prioritization scheme within a culling ring. In our results this proved to be quite efficient (as shown in Table 2), primarily because most farms that are selected in a risk based prioritization scheme are located within a ring of 3 km from an infected farm (Table 2).

In the past mainly ring culling strategies have been considered in practice and literature [3,4,20,22]. In [20,22] a strategy was modelled that prioritised farms with high probability of infection. In this work the probability of infection per farm was based on the distance to the infected farms weighted by the number of sheep and cows. In risk based culling selection of farms is done by combining the probability of infection (dependent on distance to the infected farms) with the reproduction number (dependent on the local density of farms). Our results demonstrate that including the local density of farms to determine the order of preventive culling to control an epidemic is a promising strategy. This paper provides a guideline that could help improve the effectiveness of culling.

## Competing interests

The authors declare that they have no competing interests.

## Authors' contributions

DB, JS, MK, MB conceived the study, TH, MB, DB formulated the model equations, DB programmed the model, carried out the simulations, and drafted the manuscript. All authors read, amended and approved the final manuscript.

## Supplementary Material

Additional file 1 **Supplementary text: Description of random map generation**. Click here for file

Additional file 2 **Figure S1: Border corrected Ripley's K for random maps**. Click here for file

## References

[B1] Stegeman A Elbers AR Smak J de Jong MC Quantification of the transmission of classical swine fever virus between herds during the 1997-1998 epidemic in The Netherlands Prev Vet Med 1999 42 219 234 10.1016/S0167-5877(99)00077-X 10619157

[B2] Capua I Marangon S The avian influenza epidemic in Italy, 1999-2000: a review Avian Pathol 2000 29 289 294 10.1080/03079450050118403 19184817

[B3] Ferguson NM Donnelly CA Anderson RM The foot-and-mouth epidemic in Great Britain: pattern of spread and impact of interventions Science 2001 292 1155 1160 10.1126/science.1061020 11303090

[B4] Keeling MJ Woolhouse ME Shaw DJ Matthews L Chase-Topping M Haydon DT Cornell SJ Kappey J Wilesmith J Grenfell BT Dynamics of the 2001 UK foot and mouth epidemic: stochastic dispersal in a heterogeneous landscape Science 2001 294 813 817 10.1126/science.1065973 11679661

[B5] Bouma A Elbers ARW Dekker A de Koeijer A Bartels C Vellema P van der Wal P van Rooij EMA Pluimers FH de Jong MCM The foot-and-mouth disease epidemic in The Netherlands in 2001 Prev Vet Med 2003 57 155 166 10.1016/S0167-5877(02)00217-9 12581598

[B6] Stegeman A Bouma A Elbers AR de Jong MC Nodelijk G de Klerk F Koch G van Boven M Avian influenza A virus (H7N7) epidemic in The Netherlands in 2003: course of the epidemic and effectiveness of control measures J Infect Dis 2004 190 2088 2095 10.1086/425583 15551206

[B7] Claas EC Osterhaus AD van Beek R De Jong JC Rimmelzwaan GF Senne DA Krauss S Shortridge KF Webster RG Human influenza A H5N1 virus related to a highly pathogenic avian influenza virus Lancet 1998 351 472 477 10.1016/S0140-6736(97)11212-0 9482438

[B8] Fouchier RA Schneeberger PM Rozendaal FW Broekman JM Kemink SA Munster V Kuiken T Rimmelzwaan GF Schutten M Van Doornum GJ Avian influenza A virus (H7N7) associated with human conjunctivitis and a fatal case of acute respiratory distress syndrome Proc Natl Acad Sci USA 2004 101 1356 1361 10.1073/pnas.0308352100 14745020 PMC337057

[B9] Koopmans M Wilbrink B Conyn M Natrop G van der Nat H Vennema H Meijer A van Steenbergen J Fouchier R Osterhaus A Bosman A Transmission of H7N7 avian influenza A virus to human beings during a large outbreak in commercial poultry farms in The Netherlands Lancet 2004 363 587 593 10.1016/S0140-6736(04)15589-X 14987882

[B10] Bos ME te Beest Dennis E van Boven M van Beest Holle Mirna RDu R Meijer A Bosman A Mulder M Yonne Koopmans PG Marion Stegeman A High probability of avian influenza virus (H7N7) transmission from poultry to humans active in disease control on infected farms J Infect Dis 2010 201 1390 1396 10.1086/651663 20331380

[B11] te Beest DE van Boven M Bos ME Stegeman A Koopmans M The efficacy of personal protective equipment and oseltamivir in the H7N7 avian influenza A epidemic in The Netherlands in 2003 Emerg Infect Dis 2010 16 1562 1568 20875281 10.3201/eid1610.091412 PMC3294382

[B12] Boender GJ Hagenaars TJ Bouma A Nodelijk G Elbers AR de Jong MC van Boven M Risk maps for the spread of highly pathogenic avian influenza in poultry PLoS Comput Biol 2007 3 e71 10.1371/journal.pcbi.0030071 17447838 PMC1853123

[B13] Chen Y Paul G Havlin S Liljeros F Stanley HE Finding a better immunization strategy Phys Rev Lett 2008 101 058701 18764435 10.1103/PhysRevLett.101.058701

[B14] Britton T Janson S Martin-Löf A Graphs with specified degree distributions, simple epidemics, and local vaccination strategies Adv Appl Prob 2007 39 922 948 10.1239/aap/1198177233

[B15] Galvani AP Reluga TC Chapman GB Long-standing influenza vaccination policy is in accord with individual self-interest but not with the utilitarian optimum Proc Natl Acad Sci USA 2007 104 5692 5697 10.1073/pnas.0606774104 17369367 PMC1838447

[B16] Grenfell BT Bolker BM Cities and villages: infection hierarchies in a measles metapopulation Ecol Lett 1998 1 63 70 10.1046/j.1461-0248.1998.00016.x

[B17] Ferguson NM Donnelly CA Anderson RM Transmission intensity and impact of control policies on the foot and mouth epidemic in Great Britain Nature 2001 413 542 548 10.1038/35097116 11586365

[B18] Elbers ARW Koch G Bouma A Performance of clinical signs in poultry for the detection of outbreaks during the avian influenza A (H7N7) epidemic in The Netherlands in 2003 Avian Pathol 2005 34 181 187 10.1080/03079450500096497 16191700

[B19] Tildesley M House T Bruhn M Curry R O'Neil M Allpress J Smith G Keeling M Impact of spatial clustering on disease transmission and optimal control Proc Natl Acad Sci USA 2010 107 1041 1046 10.1073/pnas.0909047107 19955428 PMC2824282

[B20] Tildesley MJ Bessell PR Keeling MJ Woolhouse ME The role of pre-emptive culling in the control of foot-and-mouth disease Proc Biol Sci 2009 276 3239 3248 10.1098/rspb.2009.0427 19570791 PMC2817163

[B21] Tildesley MJ Keeling MJ Is R0 a good predictor of final epidemic size: Foot-and-mouth disease in the UK J Theor Biol 2009 258 623 629 10.1016/j.jtbi.2009.02.019 19269297 PMC2895684

[B22] Kao RR The impact of local heterogeneity on alternative control strategies for foot-and-mouth disease Proc Biol Sci 2003 270 2557 2564 10.1098/rspb.2003.2546 14728777 PMC1691549

