# Supplementary material for: Risk based culling for highly infectious diseases of livestock
Source: Vet Res. 2011 Jun 29;42(1):81. doi: 10.1186/1297-9716-42-81 (PMC3160900; doi:10.1186/1297-9716-42-81)
Supplement: Additional file 1 — Supplementary text: Description of random map generation. [file 1297-9716-42-81-S1.DOC]

**Additional file 1, Supplementary text. Description of random map generation.**

Random maps with prescribed densities were constructed according to the following algorithm:

1. The middle of the maps (0, 0) was chosen as starting location. This point serves as the first parent (*x_p_, y_p_*).
2. For the generation of an offspring point (*x_p+1_, y_p+1_*) an angle (*a*) was selected from: *a* ~ Uniform (0, 360) and a distance (*4)* was selected from *d* ~ Exponential (*λ*). Where *1/λ* is the mean distance from the parent, λ = λ_inner_ if parent was in the inner area and λ = λ_outer_ if the parent was in the outer area.
3. Coordinates offspring point then become: (*x_p+1_*, *y_p+1_*) = (*x_p_* + *a* sin(*4)*, *y_p_* + *a* cos(*4)*)
4. Offspring becomes new parent and new offspring is generated according to 2 and 3 until there 1000 farms in both the inner and the outer area. The process was conditional on total number of farms for the inner and outer areas.
5. If no valid point is drawn after 100 iterations (either because the inner or outer area is full or because points outside the map are generated) a new random starting location is chosen.

In the base scenario the mean distances from the parent was set as such (1/λ_inner_ = 1, and 1/λ_outer_ = 3) that created maps that were reasonably clustered (Figure 4). To analyze sensitivity to farm clustering we repeated the analysis on maps with more and less clustering but with the same number of farms in the inner and outer areas. On the maps with less clustering, farm locations were homogenous poisson distributed. On maps with more clustering distances from the parent that were drawn from exponential distributions were reduced (1/λ_inner_ = 0.25, and 1/λ_outer_ = 0.75). For each scenario (base, more clustering, less clustering) Ripley’s K was calculated as measure for clustering (Additional file 2, Figure S1). Border effects in the simulation are unavoidable but reduced because the reproduction number in the outer area is below 1 and the epidemic fades out in these areas. On maps with increased clustering it is possible for the reproduction number to be above 1 in the outer ring which increases border effects. The mean reproduction number (R) across a map changes as a result of variable degrees of clustering. Since we only want to asses the effect of clustering (and not that of an increased reproduction number, which was done separately), the hazard kernel was adjusted so that the average reproduction number across all maps was the same as in the base scenario.
