# Supplementary material for: Risk based culling for highly infectious diseases of livestock
Source: Vet Res. 2011 Jun 29;42(1):81. doi: 10.1186/1297-9716-42-81 (PMC3160900; doi:10.1186/1297-9716-42-81)

**Additional file 2, Figure S1**. Border corrected Ripley’s K averaged over 50 maps for the base scenario and for the scenarios with increased and decreased clustering. Confidence bounds indicate 0.10 and 0.90 quantiles.


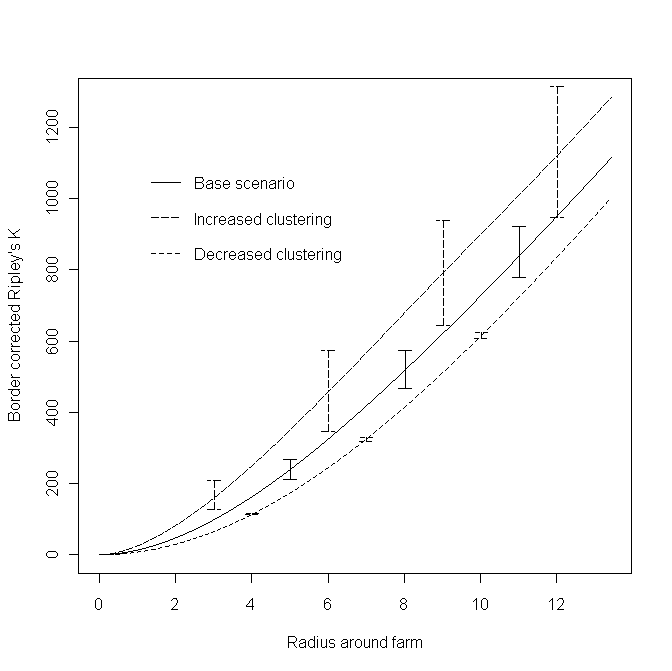

Supplement: Additional file 2 — Figure S1: Border corrected Ripley's K for random maps. [file 1297-9716-42-81-S2.DOC]
